# Undernutrition in older children and adolescents in peri-urban Zambia

**DOI:** 10.3389/fpubh.2023.1251768

**Published:** 2023-09-25

**Authors:** Shela Sridhar, Janella S. Kang, Isabel Madzorera, Ethan Zulu, Joyce Makasa, Sally Bell Cross, Davidson H. Hamer

**Affiliations:** ^1^Division of Global Health Equity, Department of Medicine, Brigham and Women’s Hospital, Boston, MA, United States; ^2^Department of Child, Youth, and Family Studies, College of Education and Human Sciences, University of Nebraska - Lincoln, Lincoln, NE, United States; ^3^Division of Community Health Sciences, School of Public Health, University of California, Berkeley, Berkeley, CA, United States; ^4^Right to Care, Lusaka, Zambia; ^5^I4life, Lusaka, Zambia; ^6^Department of Global Health, Boston University School of Public Health, Boston, MA, United States; ^7^Section of Infectious Diseases, Department of Medicine, Boston University Chobanian and Avedisian School of Medicine, Boston, MA, United States; ^8^Friedman School of Nutrition Science and Policy, Tufts University, Boston, MA, United States

**Keywords:** adolescent, malnutrition, undernutrition, adolescent malnutrition, Zambia malnutrition, wasting

## Abstract

**Background:**

Adolescents make up roughly a quarter of the population in Zambia; however, most nutrition-related programming is targeted at the under-five population. Understanding the scale of undernutrition in older children and adolescents is fundamental to alleviating food insecurity and addressing undernutrition across all age groups.

**Methods:**

A cross-sectional survey was performed in four low-income, peri-urban compounds in Chilanga District which included anthropometric measurements of children between ages 6 months-19 years and a household-level diet diversity and food security questionnaire. Wasting was used for children under 5 and thinness for children 5–19 years. Descriptive analysis and multivariate logistic regression were conducted to quantify the prevalence and distribution of malnutrition and understand the impact of food security.

**Results:**

We surveyed 393 households and 1,004 children between the ages of 6 months and 19 years. Children aged 6–9 years had the highest prevalence of severe thinness (5.2%) and adolescents (10–19 years) had the highest rates of moderate thinness (6.5%). Across all age groups, more than 75% of children were in households that worried about running out of food in the previous month. 24.9% of adolescents and 28.4% of older children were in households were more likely to go a whole day without eating compared to 16.9% of children under 5.

**Conclusion:**

Our survey indicated that malnutrition in adolescents and older children living in Chilanga district was comparable to those under 5. Interventions to address undernutrition must be targeted at older children and adolescents in order to ameliorate this burden.

## Introduction

There are roughly 1.8 billion adolescents worldwide, 90% of whom live in low- and middle-income countries (LMICs) (1). In Zambia, adolescents (aged 10–19 years) comprise about 25% of the population (2). While there have been multiple calls in recent years to further understand the impact of adolescent undernutrition (1, 3, 4), most research and programming on a global scale has focused on the under 5 (U5) population (4, 5). The importance of malnutrition in older children and adolescents has come into greater focus over the last several years given that the importance of this population is a crucial demographic to maintain a healthy society and workforce (1).

Adolescence is an important period of growth and development during which children have specific health and nutritional needs (1). Adequate nutrition is essential for several key growth and development parameters that occur during adolescence, including puberty, linear growth (which has its first peak in the first 2–3 years of life and then again in adolescence), and weight gain. During adolescence, children gain 50% of their adult weight, 40% of their peak bone mass (1) and 15–20% of their height (6). In addition to growth, the nutritional status of adolescents has notable impacts on long-term cardiovascular fitness, risk of non-communicable diseases and immunity (4) which affects the overall health indicators of a population over time. Furthermore, significant cognitive development occurs during adolescence (4) which has long-lasting impacts on their choices and their economic status into adulthood. Adequate nutritional intake is critical not only for the growth of these adolescents, but for their future families as their own health status will have significant intergenerational impacts on the nutritional well-being of their children and families (4, 7).

Globally, more than one-third of women are married by the age of 18 (1) and more than 20% of births to adolescent mothers occur in sub-Saharan Africa and Latin America. One study showed that more than 25% of mothers between the ages of 15–19 met criteria for stunting (1), a marker for poor nutrition (6). Pre-pregnancy stunting in adolescent girls is especially important given its association with pre-term birth and low birth weight, conditions which ultimately perpetuate a multigenerational cycle of undernutrition (1, 4). In Zambia, the 2018 Demographic and Health Survey (DHS) found that 29% of adolescent girls (15–19 years) have begun childbearing. This is particularly relevant in women who live within the lowest wealth quintile because 46% of these women have already begun childbearing in their adolescence (2).

Despite the noted importance of nutrition in child and adolescent health, there have been few studies quantifying the prevalence of malnutrition or undernutrition among older children and adolescents in sub-Saharan Africa, particularly in Zambia (5, 6, 8, 9). Moderate and severe thinness among children and adolescents globally is 8.4% in girls and 12.4% in boys (1). While there have been significant strides in U5 malnutrition rates, older children and adolescents are often neglected. One study from Uganda showed that the prevalence of thinness was higher for children 5–17 years compared to U5 children (5.5% vs. 3.8%) (5). This can largely be attributed to the limited availability of nutrition programs and interventions for children over 5 years (5).

While much of the mortality and economic hardship is based on U5 data, there is likely an unreported degree of malnutrition in children over 5 years. Currently, no global targets have been identified for adolescent undernutrition (4). Given the paucity of research into the adolescent population, we aimed to provide a descriptive analysis of the prevalence of adolescent malnutrition as compared to other pediatric age groups in Chilanga district within Lusaka, Zambia. We also evaluated the rates of stunting, thinness, and obesity across age groups in Chilanga, the characteristics of these groups and the impact of food security on nutritional status.

We hypothesize that the rates of undernutrition, specifically thinness and stunting, are as prevalent in older children and adolescents as in children U5, and therefore interventions must be targeted at this population. By understanding the prevalence of malnutrition in older children (6–9 years) and adolescents within the community, we can develop effective intervention strategies to address food insecurity and malnutrition. This knowledge is crucial for implementing interventions and scaling policies to address malnutrition and hunger in all forms (10).

## Methods

We conducted a cross-sectional survey of households in the Chilanga district of Lusaka Province in Zambia between February 1st and February 14, 2022. I4life, an Irish-based, non-governmental organization (NGO), focused on community-based nutrition care, was the implementing organization for this survey. They are based locally in Chilanga district and work closely with this community; therefore, we chose this location to better understand the population being served. Ethical approval was provided by the University of Zambia (UNZA) Biomedical Research Ethics Committee and the Brigham and Women’s Institutional Review Board. Logistical support was provided by Right to Care Zambia.

### Study site and population

This study was conducted in 4 compounds (neighborhoods) within Chilanga district: Freedom, Kazimva, Linda, and Mwembeshi. Chilanga district has a population of roughly 107,000 people, with about 32,000 under the age of 9 years and 26,500 between the ages of 10–19 (11). The study population included 1,004 children aged 6 months to 19 years who lived in Chilanga district. This age range was used to group children based on the WHO categorizations of childhood: under 5, older child (age 6–9 years), and adolescent (10–19 years). The full range of childhood and adolescent ages were included in order to assess differences in the prevalence of malnutrition across the three age categories.

### Sampling procedure and data collection

Within the district, households from the four different compounds were sampled. Purposive sampling was chosen to identify streets to sample within each compound because publicly available documents were not available to ascertain an accurate household number in the district and therefore, we did not have an accurate population size to define our household level population. Furthermore, we aimed to ensure that vulnerable individuals such as non-traditional families and unhoused or insecurely housed individuals were included in our data set as these are likely the populations who will likely benefit from future interventions. However, within each area of the neighborhood, the data collectors used a systematic randomized sample approach and identified every third house within a neighborhood and households were only included if they had at least one child aged 6 months to 19 years. Each data collector pair was responsible for a different region of the neighborhood to avoid double counting. Data collectors were instructed to ensure that at least 50% of their households had 1 child greater than 10 years of age. Households of data collectors within the community were excluded. Based on data collectors’ financial and time constraints, we aimed to survey 100 households in each compound. I4life staff familiar with the area identified the compounds to be included *a priori* based on their assessment of greatest nutritional need in the community. The four compounds had an even number of households.

Local and district approvals were obtained for community engagement. Informed consent was obtained from each household. Consent forms were provided in either Nyanja or Bemba (depending on the needs of the interviewee) and read to the participant in the relevant language if the participant did not have the necessary literacy level to read the form. Data collectors were members of the community fluent in the local language. They were trained on how to identify households, conduct the questionnaire, and perform anthropometric measurements. An adult in the household, typically the mother, responded to the household diet questionnaires. This questionnaire, which included the food security component was adapted from a questionnaire developed by the Africa Research Implementation Science Education (ARISE) research group to evaluate the impact of COVID-19 on adolescent health in several sub-Saharan African countries (12).

Trained research assistants collected anthropometric data, including height for children over 2 and length for children under 2 (in centimeters), weight (in kilograms), and mid-upper arm circumference (MUAC; in millimeters) of each child in the home, along with a household diet questionnaire. Anthropometric data was collected using standardized MUAC measuring tapes and UNICEF height boards for children under 5 and for children over 5 a standard tape measure was utilized. A standardized UNICEF standing electronic scale was used to obtain weights. For children unable to stand, the mother held the child on the scale. The scale was able to be zeroed out if the mother was on the scale. If a child was not present (i.e., they were in school), data collectors returned after the school day had ended to collect the data. All anthropometric measures were calculated into z-scores using the WHO Child Growth Standards STATA igrowup package for 0–5 years (13) and WHO AnthroPlus for 5–19 years (14).

### Data management

Study data were collected and managed using REDCap (Research Electronic Data Capture, Vanderbilt University, Nashville, Tennessee, United States), a secure web application for building and managing online surveys and databases hosted at Brigham and Women’s Hospital (15). Paper surveys were available in English and Nyanja for data collectors to use in case of technical challenges. Completed paper surveys were subsequently input into REDCap manually. No identifiers were obtained during data collection.

Categories of nutritional status were based on standard WHO categorizations: severe wasting or thinness, moderate wasting or thinness, moderate and severe stunting, normal nutritional status, overweight and obese (16). Wasting was defined using weight-for-height z-scores (WHZ) for U5 children and thinness was defined using body mass index (BMI) for age for children aged 6–19 years. WHO growth standards were used to calculate z-scores (13, 17). Children under 5 were considered to be moderately wasted with a WHZ between of <−2 and ≥ −3 and were characterized as severely wasted if their WHZ was less than −3. We also calculated the BMI-for-age for children under 5 for consistency with the definition for older children and adolescents. There was no difference noted in the number of children qualifying as moderate of severe thinness. Therefore, in order to maintain comparability with other studies, we presented the data using the WHZ criteria for children under 5 in our results section. The outliers were cut off using WHO standards (13, 17), where extreme values of WHZ, i.e., z-score larger than 5 or less than −6, were considered as missing variables.

WHO Child Growth Standards for children aged 6–19 years were used to calculate BMI-for-age z-scores (17). BMI z-scores between −2 and − 3 were considered moderate thinness and less than −3 were severely thin for children aged 6–19 years. All children with a height-for-age z-score (HAZ) between −2 and − 3 were considered moderately stunted and less than −3 were considered severely stunted. Overweight children aged 6–19 were defined as a BMI-for-age z-score greater than +1 and less than or equal to +2 and obesity was defined as a z-score greater than +2. For children U5, overweight was defined as a BMI-for-age z-score greater than +2 and smaller or equal to 3 and obese as BMI-for-age z-scores larger than +3 (18). Following WHO standards (13, 17), BMI-for-age z-scores larger than 5 or less than −5 were considered as outliers and not included in the analysis. Missing data was also excluded from data analysis. Similarly, HAZ larger than 6 or less than −6 were considered as outliers.

### Statistical analysis

The primary outcomes were the number of households in which older children or adolescents met the criteria for moderate or severe thinness and/or stunting. Secondary outcomes included factors associated with thinness in this population.

A descriptive analysis of nutritional status was conducted using the WHO guidelines for z-score by BMI for children over 5 and WHZ for children U5. For categorical variables, frequencies and prevalence for categorical variables and means and standard deviation (SD) for continuous variables were used to summarize population and household demographics. About 70% of the children included in the survey had birth dates included; the other 30% did not. We used DHS data imputation methodology to impute the relevant birthdates (19). A sensitivity analysis was performed between the data sets including imputed dates and the data set without dates which showed no statistically significant difference in mean ages between the 2 data sets (Welch’s *t*-test, *p* = 0.10). The full imputed data set was used for the remainder of the data.

Multivariate logistic regression, controlling for clustering by household, was used to investigate the impact of several factors on undernutrition among children aged 6 months to 19 years old. Our models include: age (U5, 6–9 years old, 10–19 years old), sex (male/female), HIV positive status (no/yes), school absences (no/yes), respondent’s occupation (unemployed, business owner, wage employment, self-employment, casual worker, and other), monthly household income (less than K1000, K1000-2400, over K2500), worry about running out of food in the past month (no/yes), skipped a meal in the past month (no/yes), went without eating for a whole day in the past month (no/yes), and received any assistance in the past month (no/yes). We selected potential confounders based on the association from the univariate regression models at *p* < 0.25. Thus, variables including access to clean water and soap, HIV status, school absences, and receiving any assistance in the past month were not included in the final regression model. Although sex and household income had a larger value of p than 0.25, these variables were included as various literature has shown the significant associations (20, 21). Odds ratios (OR) for all variables were determined using a 95% confidence interval (CI).

Data were analyzed with STATA 17.0 software. A significance level of *p* = 0.05 was used for all statistical tests and analyses.

## Results

A total of 393 households and 1,004 children were surveyed. The average household size was 5 people and 93% of households had access to water. Approximately 89.2% of households made less than 2,400 kwacha (about 135 USD in February 2023) per month (Table 1). Only 3.1% of respondents of the household survey had completed any tertiary education. Slightly over half (52.6%) of the children sampled were female (Table 1). 8.8% of children across all age groups were categorized as either severely or moderately underweight. One-third (33.4%) of all children were categorized as moderately or severely stunted.

**Table 1:** Socio-demographic characteristics of study households.

	Total			
	*n*	%	Mean	SD
Total children	1,004	–	–	–
Sex			–	–
Male	479	47.2		
Female	537	52.6		
Number of HIV positive children	18	2.0	–	–
Number of children with a positive TB history	1	0.1	–	–
Number of children with any past medical history^a^	31	3.0	–	–
Number of children with a history of hospitalization for malnutrition	12	1.2	–	–
Severe thinness across all age groups^b^	31	3.6	–	–
Moderate thinness across all age groups^b^	45	5.2	–	–
Normal weight	639	73.3	–	–
Overweight/obese	75	8.6	–	–
Moderate stunting	180	20.0	–	–
Severe stunting	119	13.4	–	–
Total households (*n* = 393)				
Number of family members in household	–	–	5.6	0.11
Presence of any family member with HIV	77	19.6	1.2	0.02
Presence of any family member with TB	17	4.4	1.0	0.01
Access to clean water	365	93.1	0.9	0.01
Role of respondent (*n* = 391)				
Mother	304	77.8	–	–
Father	38	9.7	–	–
Grandparent	16	4.1	–	–
Child	19	4.9	–	–
Other	14	3.6	–	–
Income level of household (*n* = 389)				
<1,000 kwacha^c^	216	55.5	–	–
1,000–2,400 kwacha	131	33.7	–	–
>2,500 kwacha	42	10.8	–	–
Occupation of respondent (*n* = 392)				
Unemployed	109	27.8	–	–
Business owner	108	27.6	–	–
Casual worker (maid or gardener)	83	21.2	–	–
Wage employment	52	13.3	–	–
Self-employed	35	8.9	–	–
Other	5	1.3	–	–
Education level of respondent (*n* = 389)				
None	23	5.9	–	–
Some/completed primary school education	171	44.0	–	–
Some/completed secondary/high school	181	46.5	–	–
Tertiary education	12	3.1	–	–
Other (literacy class, religious school)	2	0.5	–	–
Water, sanitation, and hygiene				
Access to clean water for washing hands (*n* = 392)	369	94.1		–
Access to clean water for cooking (*n* = 392)	365	93.1		–
Water source (*n* = 393)				
Borehole	113	28.7	–	–
Communal tap	86	21.9	–	–
Protected dug well	17	4.3	–	–
Tapped running water	155	39.4	–	–
Other	22	5.6	–	–
Soap in the household (*n* = 390)				
Yes	292	74.9	–	–
No	98	25.1	–	–
Number of children in each compound (*n* = 994)				
Linda	296	29.8	–	–
Kuzimbva	263	26.5	–	–
Mwembeshi	239	24.0	–	–
Freedom	196	19.7	–	–

^a^History of medical problems include: asthma, neuro-related disorders (autism, epilepsy, and cerebral palsy), kidney problems, and sickle cell disease.

^b^Analysis were run using both BMI for age across all age groups (6 months-19 years) and WHZ in children under 5 and BMI for age in children 5-19 years and results were unchanged.

^c^18.38 kwacha = 1 USD as of Feb 4, 2022.

The prevalence of severe thinness in adolescents was comparable to wasting in U5 children (2.7% in adolescents and 2.8% in U5 children; Table 2). Children aged 6–9 years had the highest rates of severe thinness (5.2%). Adolescents had the highest proportion of moderate thinness (6.5%) compared to 4.6% in older children and 5.2% wasting in children under 5. Stunting (moderate and severe) was most prevalent in children under 5 (48.2% compared to 25.7 and 30.3% in older children and adolescents, respectively). Age categories were closely matched with 273/1022 (27.2%) of children aged U5, 320 (31.9%) were age 6–9 years, and 411 (40.9%) were aged 10–19 (Table 2). More than 75% of children with thinness in all age groups worried about running out of food in the previous month and more than 60% across the age groups stated that they had skipped at least 1 meal. Interestingly, 91/320 (28.5%) of older children and 102/411 (24.9%) of adolescents had skipped a whole day of meals compared to 73/271 (16.8%) of children under 5 (Table 2).

**Table 2:** Nutritional classification and food security for children across age categories.

	Total		Under age 5		Age 6–9		Age 10–19		
	*n*	%	*n*	%	*n*	%	*n*	%	*p*-value
Nutritional classification									
Total	1,004	–	273	27.2	320	31.9	411	40.9	
Severe wasting/thinness	31	3.6	6	2.8	15	5.2	10	2.7	0.17
Moderate wasting/thinness	45	5.2	8	3.7	13	4.6	24	6.5	0.27
Normal weight	639	73.3	137	62.8	220	76.9	282	76.6	<0.001
Overweight/obese	157	18.0	67	30.7	38	13.3	52	14.1	0.23
Moderate Stunting	180	20.0	57	25.2	57	19.0	66	17.7	0.07
Severe stunting	119	13.2	52	23.0	20	6.7	47	12.6	<0.001
Food security*									
Worry about running out of food in the last month									0.72
No	215	21.6	59	21.7	73	23.0	83	20.4	
Yes	781	78.4	213	77.0	245	77.0	323	79.6	
Skipped a meal in the last month									0.59
No	334	33.3	97	35.5	101	31.6	136	33.1	
Yes	670	66.7	176	64.5	219	68.4	275	66.9	
Went without eating for a whole day in the last month									0.55
No	736	73.5	199	73.2	229	71.6	308	75.1	
Yes	266	26.5	73	16.8	91	28.4	102	24.9	
Received any assistance in the last year									0.94
No	928	93.2	254	93.5	295	93.0	379	93.4	
Yes	68	6.8	18	6.6	23	7.2	27	6.7	

*Differences for food security questions were assessed using *χ*^2^tests of association which were all shown to have no significant difference (*p* > 0.5).

We found that there were 68 households (17.3%) with at least one child categorized as severe or moderately underweight and 196 households (49.9%) with at least one stunted child. There were 55 households (14.0%) in which there was an older child or adolescent who met criteria for moderate or severe thinness without a child U5 meeting criterion. However, there were only 11 households (2.8%) in which only the U5 child in the household met criteria, but older children and adolescents did not meet the criterion. Demographics, including income, household size and education of households with only thin older children were similar to those with only U5 children who were underweight.

Food security at the household level was similar as well. Households in which only the older child was categorized as moderately or severely thin (79.2%) were more likely to have a child who had skipped a meal in the last month compared to the other age groups (58.3% in children U5 and 65.5% in adolescents). More than 85% of households across all age groups with at least one malnourished child worried about running out of food (Table 3). Despite more households with only an adolescent meeting criterion for thinness, none of the households with only an adolescent experiencing thinness received assistance.

**Table 3:** Household level food security with thinness stratified by age group.

	Households with only U5 children with thinness		Households with only older children (age 6 to 9 years) with thinness		Households with only adolescents (age 10 to 19 years) with thinness	
	*n* = 12	%	*n* = 24	%	*n* = 29	%
Worry about running out of food (*n* = 387)						
No	1	8.3	3	12.5	4	13.8
Yes	11	91.7	21	87.5	25	86.2
Skipped a meal (*n* = 389)						
No	5	41.7	5	20.8	10	34.5
Yes	7	58.3	19	79.2	19	65.5
Went without eating for a whole day (*n* = 388)						
No	8	66.7	18	75.0	23	79.3
Yes	4	33.3	6	25.0	6	20.7
Received any assistance (*n* = 387)						
No	11	91.7	22	91.7	29	100.0
Yes	1	8.3	2	8.3	0	0.0

There was an inverse relationship between stunting and thinness (OR 0.39, 95% CI 0.20 to 0.76, *p* < 0.05; Table 4). We also did not find significance in relationship between thinness and monthly household income or parent occupation as we expected. Similarly, we expected to find an association between food security and thinness, but no association was noted.

**Table 4:** Univariate and multivariate logistic regression for factors associated with thinness in Chilanga district among children age 6 months to 19 years.

	Univariate		Multivariate	
	OR	95% CI	OR	95% CI
Age				
<5 years	ref			
Age 6–9 y	1.58	0.81, 3.08	1.08	0.53, 2.20
Age 10–19 y	1.48	0.78, 2.83	0.96	0.46, 1.35
Sex				
Male	ref			
Female	0.86	0.54, 1.37	0.79	0.46, 1.35
Stunting	0.37*	0.19, 0.69	0.31*	0.15, 0.66
Occupation				
Unemployed	ref			
Business owner	1.16	0.47, 2.85	0.85	0.27, 2.68
Wage employment	0.57	0.07, 4.98	0.92	0.09, 9.26
Self-employed	0.70	0.23, 2.08	0.82	0.23, 2.94
Causal worker (maid or gardener)	1.22	0.47, 3.18	1.38	0.44, 4.30
Other	0.96	0.35, 2.66	1.05	0.31, 3.50
Household income				
Less than K1000	ref			
K1000–K2400	0.82	0.49, 1.38	0.95	0.53, 1.73
K2500 and above	0.73	0.32, 1.67	0.73	0.26, 2.09
Worry about running out of food				
No	ref			
Yes	1.78	0.92, 3.44	1.27	0.54, 2.97
Skipped a meal				
No	ref			
Yes	1.44	0.85, 2.42	0.94	0.44, 2.00
Went without eating for a whole day				
No	ref			
Yes	1.55	0.94, 2.56	1.67	0.89, 3.16
Received any assistance				
No	ref			
Yes	0.55	0.17, 1.80	0.47	0.11, 2.05

*p < 0.05, **p < 0.01.

## Discussion

In Chilanga district, a relatively low socio-economic community in Zambia, we found that the prevalence of undernutrition in the adolescent population was similar to or even slightly higher than that of the traditionally measured U5 population. While our sample was generally comparable to national nutritional data, we found a slightly higher prevalence of stunting and wasting in Chilanga district in the under 5 population when compared to national DHS data. 48.2% of our population was stunted compared to 35% of the national population and 4 % of the national population is characterized as wasted, whereas 8.8% in our population were wasted (2). Of note, the DHS does not quantify the rates of undernutrition in older children or adolescents, which we found to have an equal or even greater need for nutritional intervention; however, due to insufficient published data, we are unable to compare to the national need of these two age groups to the findings in our study.

It bears noting that this survey was also conducted immediately after the Omicron surge of the COVID-19 pandemic. The pandemic has significantly impacted food security and malnutrition globally (22, 23) and higher rates of malnutrition that we saw may also present nationally, but have not yet been quantified. These higher rates of malnutrition will likely persist for the next several years given the long-term ramifications of public health policies and health effects of the pandemic (22).

The inverse relationship between stunting and thinness was a surprising finding as it does not correlate with the broader literature. This may have been related to the higher incidence of obesity in the U5 population relative to the older age groups given that the U5 population also had the highest rate of stunting. This difference in stunting rates between these two age groups is also unclear as these children are from similar families and locations. It was considered that this difference was due to the impact of the COVID-19 pandemic and the higher incidence of food insecurity and malnutrition globally in the two-year period preceding this survey. However, none of the food security indicators were associated with stunting in our models. This may be related to the small sample size, or given that there were 393 households with 1,004 children total, many children would have come from the same house resulting in a relatively homogenous population which was not accounted for. Additional investigation should be pursued.

In particular, we did not capture whether an adolescent might also be the mother of a young child in some multigenerational homes. In our survey, the majority of the study population made less than 150 USD per month which is roughly on par with the national income *per capita* in Zambia of 1,120 USD per year (24). The majority of households (78.4%) worried about not having enough food and 66.7% had skipped a meal in the previous month. Given this, it is likely that many children are not receiving adequate nutrition at home. One potential opportunity would be to explore the utility of food programs within schools. While private schools may be equipped with such programs, these programs do not exist in the public or government school system in Zambia. While data are mixed regarding the effectiveness of school feeding programs, some evidence suggests that in children with moderate thinness, there is an improvement in height, cognition, and school feeding programs (25). Interestingly, there did not appear to be a direct correlation with adolescent malnutrition in the household with the number of children in the family as might be expected. Understanding these factors further is crucial to developing effective interventions within these communities.

Our study has many strengths. This is one of the few studies we identified which has quantified the prevalence of adolescent malnutrition in Zambia. This is a novel survey since it evaluated the prevalence of adolescent undernutrition when compared to younger children within the same household. This allowed us to quantify the absolute rates and understand the epidemiology of the burden of disease within our community. This survey was adapted from a comprehensive survey including questions around water and sanitation (WASH) and food security which allowed us to understand the broader context of access within communities (23).

Our study also has a few limitations. While our findings are specific to the Chilanga district in Lusaka Province, our results are only applicable to that of a peri-urban environment and may not be generalizable to more rural communities where resources such as transportation or access to food, education, and work may differ. However, given that in Zambia there are higher malnutrition rates overall in rural areas, we anticipate that these findings may be even more pronounced in those settings.

Additionally, our assessment of predictors of undernutrition did not reveal any significant risk factors. This may be due to the relative homogeneity of our population as noted above. Given our smaller sample size, we did not perform an initial cluster analysis to account for this homogeneity. This may also contribute to being underpowered to make further generalizations. While this survey was intended to inform local communities about the current epidemiology in their community, expanding this survey to a larger, randomized group may be helpful in further elucidating the factors associated with thinness among older children and adolescents as we were likely underpowered to make more general conclusions. Furthermore, while our sensitivity analysis demonstrated minimal difference when we included imputed data, our data are somewhat limited as only 726 (69.1%) of the participants had included dates of birth and the rest were imputed using the DHS method.

The findings of our survey are notable as much of the current policies and programming target children under 5. While the U5 population is undoubtedly a critical cohort since the majority of the direct health costs associated with malnutrition occur in the first year of life (26), in a context in which almost 30% of adolescent females have a child (2), addressing malnutrition in this age group will have long lasting beneficial effects. Additionally, as children age and experience persistent thinness and stunting, they receive less schooling and ultimately are not able to contribute to the local workforce (26) which has long-term impacts on the perpetuation of poverty and the health and well- being of a society.

## Conclusion

Further research into undernutrition related to older children and adolescents is crucial as the drivers of poor nutrition and ultimately interventions to address this issue differ in this population compared to the U5 children. Adolescents in particular have more choice of what they eat (27) and many may have their own families to feed (28). A multidisciplinary approach to addressing this burden is necessary along with greater global investment (4).

Our survey was a first step in identifying and quantifying this burden of disease in Chilanga district; however larger scale surveys should be conducted in varied settings across the country to further understand the burden of adolescent malnutrition. The burden of undernutrition in the Chilanga district is a significant issue across childhood and adolescents, not only in U5 children. Additionally, households with malnourished adolescents in our cohort received the least assistance. This suggests that additional focus should be placed on older children and adolescent populations, both for further research to understand the scope of the problem and drivers of undernutrition as well as to design relevant interventions that address the perpetuation of malnutrition.

## Data availability statement

The raw data supporting the conclusions of this article will be made available by the authors, without undue reservation.

## Ethics statement

The studies were approved by Brigham and Women’s Hospital Institutional Review Board and the University of Zambia Ethical Review Board (UNZAREC). The studies were conducted in accordance with the local legislation and institutional requirements. Written informed consent for participation in this study was provided by the participants’ legal guardians/next of kin.

## Author contributions

All authors have made a substantial contribution to the concept or design of the article, or the acquisition, analysis, or interpretation of data for the article. All authors have drafted the article or revised it critically for important intellectual content and approved the version to be published. All authors agree to be accountable for all aspects of the work in ensuring that questions related to the accuracy or integrity of any part of the work are appropriately investigated and resolved.
